# Macular hole retinal detachment after intravitreal Conbercept injection for the treatment of choroidal neovascularization secondary to degenerative myopia: a case report

**DOI:** 10.1186/s12886-019-1164-4

**Published:** 2019-07-22

**Authors:** Chuan-bin Sun, Yueye Wang, Shiyang Zhou, Xudong Fang, Danni Xu, Zhe Liu

**Affiliations:** 1 Eye Center, Second Affiliated Hospital of Zhejiang University School of Medicine, Hangzhou, 310009 China; 2 Department of Ophthalmology, Zhejiang Provincial People’s Hospital, People’s Hospital of Hangzhou Medical College, Hangzhou, 310015 China

**Keywords:** Choroidal neovascularization, Macular hole, Conbercept, Intravitreal injection, Degenerative myopia

## Abstract

**Background:**

We report a case of macular hole (MH) formation and retinal detachment after intravitreal conbercept injection for the treatment of choroidal neovascularization (CNV) secondary to degenerative myopia.

**Case presentation:**

A 60-year-old woman presented with blurred vision in her left eye was diagnosed as CNV secondary to degenerative myopia. Intravitreal injection of conbercept, an anti -vascular endothelial growth factor (VEGF) agent, was uneventfully performed in the left eye. Unfortunately, a full thickness MH and retinal detachment was found three weeks postoperatively by ophthalmoscopy and spectral-domain optical coherence tomography. Vitrectomy, internal limiting membrane peeling and silicone oil tamponade were then performed, and macular retina was reattached soon after surgery. However, MH still kept open during three months’ follow-up.

**Conclusion:**

MH is a quite rare complication of intravitreal anti- VEGF agent injection, tangential contraction secondary to CNV shrinkage and regression caused by anti-VEGF agent is proposed to be the major pathogenesis of MH formation.

## Background

Choroidal neovascularization (CNV) is characteristic of many sight-threating fundus diseases such as exudative age-related macular degeneration (AMD), polypoidal choroidal vasculopathy, myopic CNV, and idiopathic CNV [1–3]. Recently, intravitreal anti-vascular endothelial growth factor (VEGF) agents have successfully improved the treatment outcome and visual prognosis of CNV. However, macular hole (MH) formation has emerged to be a new challenging adverse effect of intravitreal anti-VEGF agent therapy for CNV, which was mostly reported in anti-VEGF therapy for exudative AMD cases [3–13].

We herein present a case of MH formation and retinal detachment after intravitreal conbercept injection for the treatment of CNV caused by degenerative myopia.

## Case presentation

**Fig. 1:**
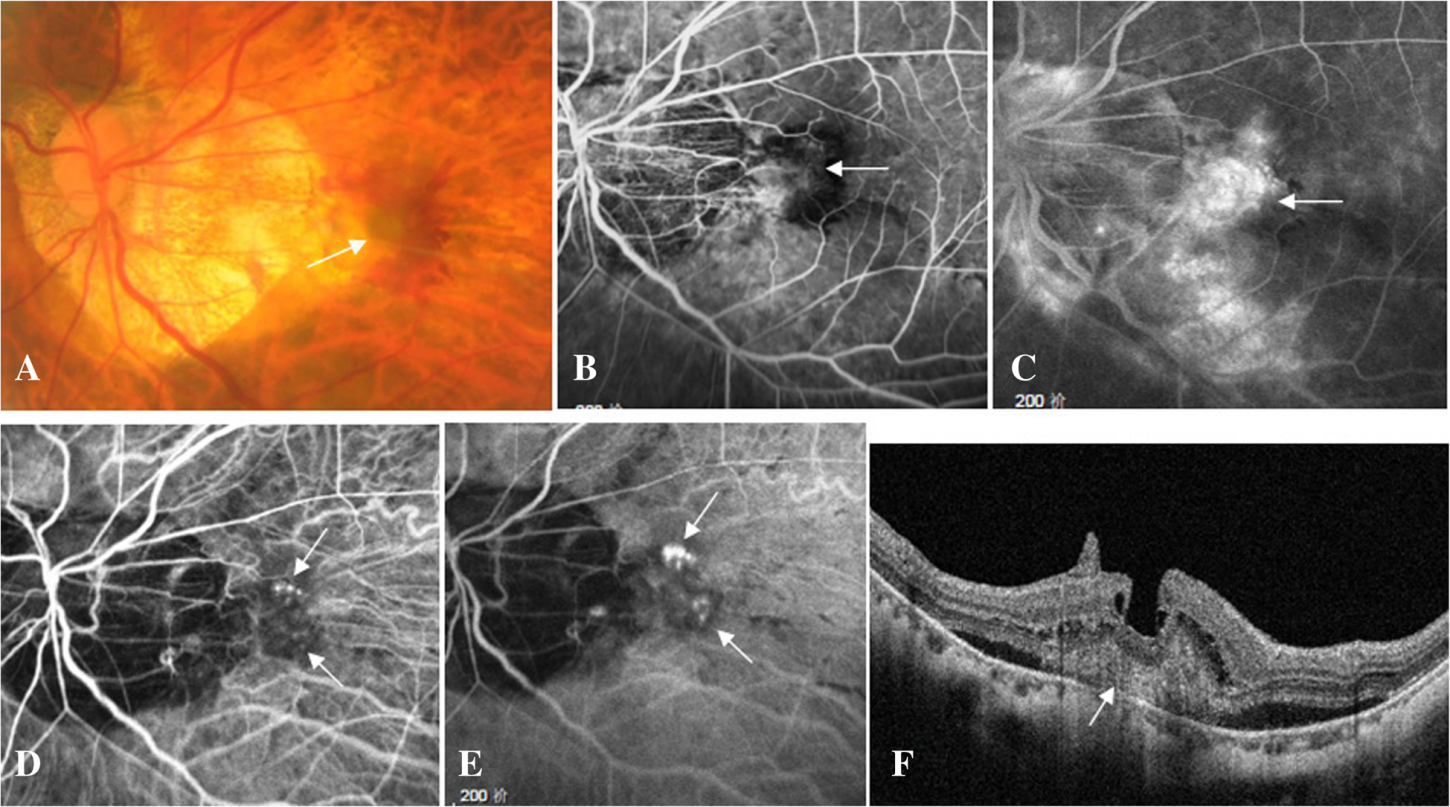
Macular findings on presentation. A 60-year-old woman presented with a complaint of blurred vision in her left eye for one week. On presentation, the best corrected visual acuity (BCVA) of her left eye was hand motion, the axial length 30.43 mm. Color fundus photograph showed grey submacular membrane (arrow) surrounded by subretinal hemorrhage (**a**). Fundus fluorescein angiography revealed early-staged submacular hyperfluorescence lesion (arrow, **b**), followed by strong fluorescein leakage and enlargement of hyperfluorescence in the late phase (arrow, **c**). Indocyanine green angiography demonstrated early-staged clustered hyperfluorescence spots (arrow, **d**), which showed evident leakage and enlargement in the late phase (arrow, **e**) . Spectral-domain optical coherence tomography (SD-OCT) revealed type 2 CNV (arrow) surrounded by serous neurosensory macuar detachment and intraretinal cysts above CNV (**f**)

A 60-year-old woman complained of blurred vision in her left eye for one week. On presentation, the best corrected visual acuity (BCVA) was 0.6 in the right eye, and hand motion in the left eye. Slit lamp examination revealed normal anterior segment in both eyes. Direct ophthalmoscopy revealed slight macular epiretinal membrane in the right eye, and grey submacular membrane surrounded by subretinal hemorrhage in the left eye (Fig. 1a). The axial length of the right eye was 30.61 mm, and that of the left eye was 30.43 mm by IOL Master measurement. Fundus fluorescein angiography revealed early-staged submacular hyperfluorescence lesion (Fig. 1b), followed by strong fluorescein leakage and enlargement of hyperfluorescence in the later phases (Fig. 1c). Indocyanine green angiography demonstrated early-staged clustered hyperfluorescence spots (Fig. 1d), which showed evident leakage and enlargement in the later phases (Fig. 1e), confirming an active CNV in the left eye. Spectral-domain optical coherence tomography (SD-OCT) revealed type 2 CNV surrounded by serous neurosensory macuar detachment and intraretinal cysts above CNV in the left eye (Fig. 1f). Her past medical history was not remarkable. CNV secondary to degenerative myopia in the left eye was then diagnosed and uneventful intravitreal conbercept (2.5 mg/0.05 ml) injection was performed soon after the informed consent was signed.

**Fig. 2:**
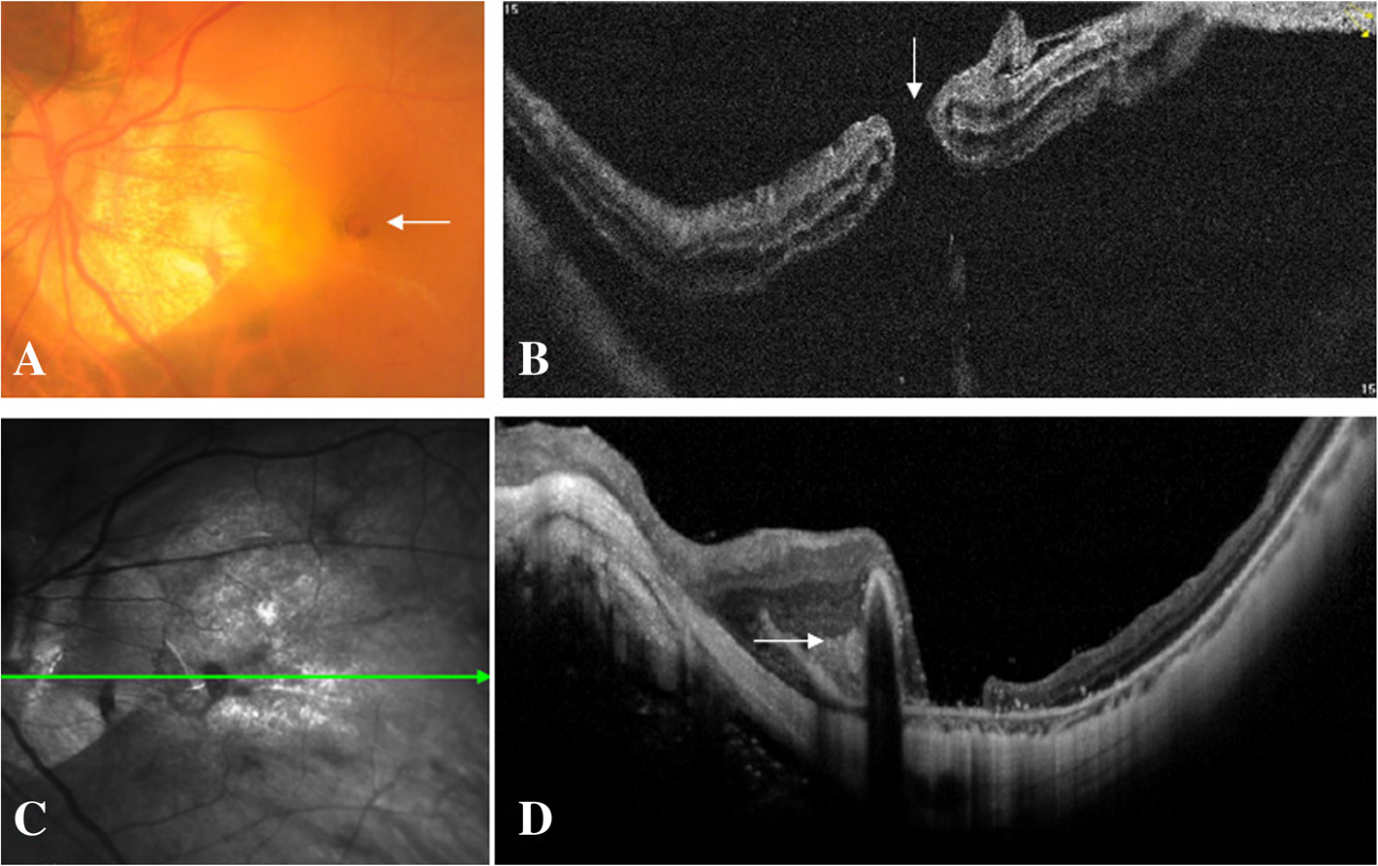
Macular hole formation after intravitreal conbercept injection. Three weeks after the injection, BCVA was counting fingers in the left eye, color fundus photograph revealed a full thickness macular hole (MH) and surrounded retinal detachment (arrow, **a**). SD-OCT comfirmed a full thickness MH (arrow) accompanied by macular detachment and intraretinal cysts in the left eye (**b**). Three months after the vitrectomy and silicone oil tamponade, BCVA was 0.05, color fundus photograph demonstrated that MH still kept open despite successful macular reattachment and Fuchs spot formation which was characterized by a hyperreflective subretinal spot in SD-OCT (arrow, **c**, **d**)

Three weeks after the injection, the patient came back with a complaint of central scotoma in the left eye. BCVA was counting fingers, funduscopic examination revealed a full thickness MH and surrounded retinal detachment (Fig. 2a), SD-OCT comfirmed a full thickness MH accompanied by macular detachment and intraretinal cysts in the left eye (Fig. 2b). Vitrectomy, internal limiting membrane peeling assisted by indocyanine green staining, and silicone oil tamponade were successfully performed in the left eye. Postoperative SD-OCT at one week’s follow-up revealed a reattached macular retina yet still open MH in the left eye. At three months’ follow-up, BCVA in the left eye was 0.05, MH still kept open depite successful macular reattachment and Fuchs spot formation (Fig. 2c, d) which was characterized by a hyperreflective subretinal spot in SD-OCT.

## Discussion and conclusions

MH is a rare yet sight threating complication of intravitreal anti-VEGF agent injection [3–13]. Our literature research based on Pubmed database revealed that there were only dozens of cases who developed a full thickness MH after intravitreal injection of anti-VEGF angents which were mostly involved with ranibizumab and bevacizumab, and occasionally with aflibercept [3–15]. To our knowledge, this is the first case report of MH formation after intravitreal conbercept injection.

Our report about conbercept-induced MH formation, together with previous case reports of other anti-VEGF agents (such as ranibizumab, bevacizumab, and aflibercept) induced MH formation, provides an important insight into the pathogenesis of MH formation after anti-VEGF therapy in eyes with CNV, and reveals that MH formation would be caused by anti-VEGF effect rather than the other characteristics of anti-VEGF agents such as molecular weight, pH, and three- dimensional structure. This finding is of great importance in helping CNV patients to choose an appropriate anti-VEGF agent.

The exact pathogenesis of MH formation after intravitreal injection of anti-VEGF angents is still under discussion. Shrinkage and regression of CNV induced by anti-VEGF agents could cause centrifugal tangential contraction, this contraction probably led to tractional forces to foveal neural retina, and finally developed a full thickness MH [4, 5, 11–15]. In this case, CNV tissue was positioned centrally beneath fovea before intravitreal conbercept injection, yet the regressed CNV tissue (Fuchs spot) was located at one edge of the MH after conbercept injection, which implied that the tangential contraction caused by the shrinked CNV might be the main mechanism of MH formation in CNV cases with intravitreal anti-VEGF agent injection. Many previous literature reports showed similar findings which strongly supported our speculation [4, 5, 11–15].

Although MH formation after intravitreal anti-VEGF agent injection is quite rare, it should be kept in mind that such complication might occur after intravitreal injection of any anti-VEGF agent. CNV Patients should be warned about this potential complication, and it should also be included in the differential diagnosis when CNV lesions did not show any therapic response to or even deteriorated after anti-VEGF agent therapy [4, 5, 13, 15].

In conclusion, MH is a quite rare complication of intravitreal anti-VEGF agent injection, tangential contraction secondary to CNV shrinkage and regression caused by anti-VEGF agent is proposed to be the major pathogenesis of MH formation.

## Data Availability

All data generated or analysed during this study are included in this published article.

## Abbreviations

AMD: Age-related macular degeneration
BCVA: Best corrected visual acuity
CNV: Choroidal neovascularization
MH: Macular hole
SD-OCT: Spectral-domain optical coherence tomography
VEGF: Vascular endothelial growth facto
